# Validity and Reliability of Short-Form McGill Pain Questionnaire-2 (SF-MPQ-2) in Iranian People with Parkinson's Disease

**DOI:** 10.1155/2020/2793945

**Published:** 2020-08-18

**Authors:** Maryam Mehdizadeh, Seyed-Mohammad Fereshtehnejad, Sepide Goudarzi, Ainaz Moshtagh, Farzaneh Dehghanian Nasrabadi, Sayed Amir Hasan Habibi, Ghorban Taghizadeh

**Affiliations:** ^1^Cellular and Molecular Research Center, Iran University of Medical Sciences, Tehran, Iran; ^2^Department of Neurosciences, Faculty of Advanced Technologies in Medicine, Iran University of Medical Sciences, Tehran, Iran; ^3^Division of Clinical Geriatrics, Department of Neurobiology, Care Sciences and Society (NVS), Karolinska Institute, Stockholm, Sweden; ^4^Division of Neurology, Faculty of Medicine, University of Ottawa, Ottawa, ON, Canada; ^5^Department of Pharmacology and Toxicology, Faculty of Pharmacy, Tehran University of Medical Science, Tehran, Iran; ^6^Department of Optometry, Faculty of Rehabilitation Sciences, Shahid Beheshti of Medical Science, Tehran, Iran; ^7^Department of Neurology, Rasoul Akram Hospital, Iran University of Medical Science, Tehran, Iran; ^8^Rehabilitation Research Center, Department of Occupational Therapy, School of Rehabilitation Sciences, Iran University of Medical Science, Tehran, Iran

## Abstract

**Purpose:**

Pain is one of the nonmotor symptoms of Parkinson's disease (PD) that, in order to be better managed, requires to be evaluated. Evaluations are done using pain assessment scales such as the Short-Form McGill Pain Questionnaire-2 (SF-MPQ-2). The goal of this study was to assess the psychometric properties of SF-MPQ-2 to measure pain in people with PD.

**Methods:**

Four hundred and twenty-eight PD patients with a mean (SD) age of 60.11 (11.44) years were included. Accessibility was measured through floor and ceiling effects. Dimensionality was estimated by exploratory factor analysis. The association between SF-MPQ-2 and other scales such as Neuropathic Pain Symptom Inventory, Douleur Neuropathic 4, Brief Pain Inventory, King's Pain Parkinson's Disease Scale, and Visual Analog Scale-Pain was considered to calculate convergent validity. Internal consistency and test-retest reliability were assessed by Cronbach's alpha and intraclass correlation coefficient (ICC), respectively.

**Results:**

A noticeable floor effect was found. Dimensionality results indicated four factors for this scale. A strong relationship was found between the SF-MPQ-2 total score and other scales (*r* = 0.55 to 0.85). In reliability analysis, Cronbach's alpha and ICC were 0.93 and 0.94 for SF-MPQ-2, respectively.

**Conclusion:**

The results of this study showed that SF-MPQ-2 has adequate validity and reliability to measure pain in people with Parkinson's disease.

## 1. Introduction

Symptoms of Parkinson's disease (PD) fall into two categories: motor (tremor, rigidity, etc.) and nonmotor (fatigue, dementia, pain, etc.), among which pain is commonly reported. According to studies, pain in patients with PD can affect their activities, consequently reducing their quality of life [1, 2]. About 30–80% of people with PD describe different types of pain, such as musculoskeletal and neuropathic pain [3, 4]. Musculoskeletal and neuropathic pain have been reported in about 70% and 20% of people with PD, respectively. Neuropathic pain includes cases caused by direct injury or disease that affect the somatosensory system [5]. The pathophysiology of this type of pain in the PD has not yet been precisely determined, but some studies have shown that it may be due to the unusual function of nociceptive processing in the central nervous system (basal ganglia-thalamocortical circuits) [6]. The clinical features of this type of pain are burning sensation with sudden flare-ups and a decrease in the sensory threshold of pain, which is associated with more involvement in the body side with predominant motor symptoms [7]. Musculoskeletal pain in these patients is mostly caused by abnormal posture/rigidity and akinesia due to motor fluctuations [7,8]. Patients with PD who had such pain do not receive adequate analgesic treatments, but some studies have shown that levodopa administration, deep brain stimulation, pain management approaches, and rehabilitation exercises can be partially effective in treating these pains [7–10].

The final purpose of therapeutic approaches recommended by researchers and clinicians to manage pain in the PD population and other diseases is to improve the quality of life [11, 12]. Moreover, to obtain an appropriate and effective therapeutic approach, pain measurement is needed to be performed via valid and accurate instruments [13].

In a recent review of the rating scales for pain in PD [14], it has been reported that King's Parkinson's disease Pain Scale is the only specific tool to measure pain in people with PD that measures different aspects of pain. On this scale, neuropathic pain and changes in the sensory threshold that lead to pain with a weaker stimulant are assessed with very few items. In contrast, Short Form-McGill Pain Questionnaire-2 (SF-MPQ-2), a modified version of the SF-MPQ, which evaluates different dimensions of pain in 22 items, is used as an appropriate and common tool for measuring pain intensity in more detail, as a gold-standard test in other populations to assess pain intensity [14, 15]. Unfortunately, the lack of psychometric properties of this questionnaire in the PD population has confined the use of this general questionnaire.

The SF-MPQ-2 has been validated in various populations (e.g., cancer, diabetes, and low back pain) and multiple languages with reports of acceptable psychometric properties [13, 15–21]. Therefore, the purpose of this study was to assess the validity and reliability of the SF-MPQ-2 in Iranian people with PD.

## 2. Material and Method

### 2.1. Participants

Four hundred and twenty-eight people with Parkinson's disease (264 men with a mean age of 60.91 years), who referred to the movement disorders clinic, were recruited in this study from July 2017 to September 2019. Inclusion criteria consisted of diagnosis of Parkinson's disease based on UK Bank Criteria [22], the ability to read and write in Persian, absence of a significant cognitive impairment (Mini-Mental Status Examination >24) [23], and absence of diseases with pain symptoms that might influence the feeling of pain, for example, severe arthritis and low back pain.

All individuals completed the written informed consent before enrollment. The study protocol was also approved by the Student Research Committee of Iran University of Medical Sciences, Tehran, Iran.

### 2.2. Procedure

After a thorough description of the study process, participants were asked to complete the SF-MPQ-2. Considering that SF-MPQ-2 is a scale used to mainly measure neuropathic and musculoskeletal pain, to reach a more accurate convergent validity, more specified tools such as Brief Pain Inventory, Neuropathic Pain Symptom Inventory, Douleur Neuropathique 4 Questions, along with King's Pain Parkinson's disease Scale, Visual Analog Scale-Pain, and Parkinson's disease Questionnaire-8 were applied. In order to solve the question of validation of these tools in PD, the psychometric properties of these three scales were assessed and analyzed as a pilot before the study, and the results were acceptable. All assessments were performed after one hour of anti-Parkinson's medication during the on-phase (one hour after taking anti-Parkinson's medication [24]). To assess test-retest reliability, 100 patients, whose medication dose was fixed for 10–14 days, were asked to refer again.

## 3. Instruments

- Short Form-McGill Pain Questionnaire-2 (SF-MPQ-2) is a revised version of the SF-MPQ scale. Participants rate the severity of their pain on a 22-item scale ranging from 0 to 10. This scale consists of four subscales of continuous, intermittent, neuropathic, and affective. The total score is obtained by summing the scores of each item; the higher the score, the greater the severity of pain. The Persian version of this questionnaire has been validated in 2014 [13, 17].
- The Neuropathic Pain Symptom Inventory (NPSI) is a 12-item scale for measuring neuropathic pain. The first 10 items and the last two evaluate the pain quantitatively and qualitatively, respectively. Scores on this scale range from 0 to 100, in which a lower scores indicates less pain [25].
- Douleur Neuropathic 4 (DN-4) is a 10-item scale to measure neuropathic pain. Each item that the patient gives a positive response is considered as one score. The maximum score of 10 means the most severe pain [26].
- Brief Pain Inventory (BPI) has two subscales of “severity” and “interference” to measure neuropathic pain. Items on this scale measure the pain in the past 24 hours with a score of 0 to 10. A higher score on this scale indicates greater pain intensity and greater disruption of daily activities [27].
- King's Pain Parkinson's Disease Scale (KPPS) is a specific scale for measuring pain in people with Parkinson's disease. It consists of 14 items. Each item score ranges between 0 and 12, obtained by multiplying each item of severity (0 to 3) and repetition (0 to 4). Total items range from 0 to 168, with lower scores indicating less pain [28].
- Visual Analog Scale-Pain (VAS-Pain) is a 100 mm linear scale with high validity and reliability for pain assessment. It ranges from “no pain” on one end to “the most severe pain” on the other. Individuals mark a point on the line according to their level of pain [29].
- Parkinson's Disease Questionnaire-8 (PDQ-8) is an 8-item questionnaire that specifically measures the quality of life in people with Parkinson's disease. Each of these items assesses one of the dimensions of the quality of life being affected. It is scored on a scale of 0 to 4 and a higher score indicates a lower quality of life. The Persian version of the PDQ-8 has been previously validated [30].

## 4. Statistical Analysis

Demographic characteristics and SF-MPQ-2 scores are described using frequency, mean, and standard deviation where appropriate. All analytical procedures were performed using IBM SPSS Statistics 22.

The sample size was calculated according to the item-to-respondent ratio of 1 : 15 [31]. Since the SF-MPQ-2 has 22 items and considering a drop-out rate of 20%, the required sample size of 396 patients was obtained. Meanwhile, as it is recommended to consider as large a sample size as possible [32], data were obtained from 428 patients with PD that was greater than the required sample size. An acceptability analysis was performed to examine missing data (less than a 5% value of calculable data was considered acceptable) and floor and ceiling effects (less than 15% of subjects who achieved the maximum or minimum score) [28].

The dimensionality of the BPI was calculated by an exploratory factor analysis (EFA) by the principal component analysis method via Varimax rotation. Kaiser-Meyer-Olkin (KMO) and Bartlett's test of sphericity were done for defining sampling adequacy. Values greater than 0.7 and *p* < 0.05, respectively, were considered sufficient. Based on Eigenvalues greater than 1 (Kaiser's criterion) and absolute loading values of 0.4 or higher, the number of factors was considered adequate [33].

Spearman correlation coefficient test was used to analyze the relationship between total score of SF-MPQ-2 and the other pain batteries, namely, DN4, VAS-P, NPSI, KPPS, and BPI. We considered a correlation coefficient of *r* > 0.60 as high and 0.30–0.59 as a moderate association [34].

Internal consistency of SF-MPQ-2 was analyzed using Cronbach's alpha coefficient where an *α* coefficient greater than 0.80 indicates high consistency. An interitem correlation was performed to examine the relationship between each item with other items (correlation higher than 0.20 was considered significant) [35].

Test-retest reliability was determined by Inter-Class Correlation (ICC) coefficient with a two-way random (absolute agreement), single measure method with a 95% confidence interval (CI). This coefficient is calculated to determine the degree of agreement between the scores measured repeatedly in different sessions for each participant. An ICC above 0.80 reflected high reliability [36]. Standard error of mean (SEM) was also calculated by the formula SEM = SD_pooled_√(1−ICC). SEM represents random variations of the score caused by repeated measurements and SEM < 1.2 SD_pooled_ was considered acceptable in this study [28].

## 5. Results

The demographic characteristics of the participants are summarized in Table 1. Accordingly, the mean (SD) age and disease duration (from the time of diagnosis) was 60.91 (11.44) and 5.39 (5.50) years, respectively. Also, the mean (SD) of the total score of SF-MPQ-2 was 9.90 (2.32) which ranged from 0 to 152. The mean (SD) of “continuous,” “intermittent,” “neuropathic,” and “affective” subscores of the SF-MPQ-2 were 3.26 (6.15), 1.31 (3.87), 3.99 (8.40), and 1.32 (3.98), respectively.

In this study, the data of 12 patients were excluded from the analysis process because they did not fully complete the questionnaire and the missing data was acceptable. The ceiling and floor effects for this scale were 0% and 60.74%, respectively.

Exploratory factor analysis demonstrated that the items of SF-MPQ-2 could be classified into four factors with a total variance of 44.78 and KMO = 0.89. The first factor includes throbbing pain, cramping pain, gnawing pain, aching pain, heavy pain, and tender items and the second factor includes shooting pain, stabbing pain, sharp pain, and splitting pain items. Electric-shock pain, piercing, cold-freezing pain, hot-burning pain, pain caused by light touch, itching, tingling or “pins and needles,” and numbness items were in the third factor and tiring-exhausting, fearful, sickening, and punishing-cruel items were in the fourth factor (Table 2).

There were moderate to high correlation between the total score of SF-MPQ-2 and DN4, VAS-P, NPSI, KPPS, and BPI scores with correlation coefficients ranging from 0.55 to 0.85, as well as a moderate correlation between the total score of SF-MPQ-2 and PDQ-8 (*r* = 0.32). A moderate-to-high correlation was also found between different subscores of SF-MPQ-2 DN4, VAS-P, NPSI, KPPS, and BPI (*r* = 0.31 to 0.85) (Table 3).

Cronbach's alpha was 0.93 which demonstrates high consistency. Also, the interitems correlation ranged from 0.21 to 0.74, indicating a satisfactory relationship between the items (Table 4). Cronbach's alphas for the “continuous,” “intermittent,” “neuropathic,” and “affective” subscales were obtained as 0.74, 0.78, 0.85, and 0.71, respectively.

In test-retest reliability, the ICC for the total score of the SF-MPQ-2 was 0.94 (95% CI = 0.89–0.97). Additionally, SEM for this scale is 4.77 which is less than 1.2 SD_pooled_.

## 6. Discussion

The purpose of this study was to evaluate the psychometric characteristics of the Short-Form McGill-2 Pain Questionnaire in subjects with PD. The results of the present study revealed that this scale has adequate validity and reliability for the assessment of pain, despite having a floor effect.

The results of our study showed that there is an unacceptable floor effect for this questionnaire. This result is in line with previous studies that have investigated the level of pain in people with Parkinson's disease via other scales [28]. This might be explained by the fact that patients with PD might experience low levels of pain in the early stages, which are not detectable with these pain measurement batteries initially.

Findings from dimensionality analysis of the SF-MPQ-2 in our PD population were in line with other disease entities demonstrating a four-factor scale, which can be used to measure pain in various dimensions, including continuous, intermittent, neuropathic, and affective [13, 16–21].

Our results also showed that there is a high correlation between the total score of the SF-MPQ-2 and other instruments that particularly assess pain. This proposes the idea that this questionnaire, along with other tools, especially the KPPS, which is a specific tool to measure pain in people with PD, could have clinical and research applications in people with Parkinson's disease [7–10]. Fewer studies, however, have examined this convergence between the total score of SF-MPQ and other pain assessment tools. A high correlation coefficient was obtained between the subscores of SF-MPQ-2 and the score of other scales, either general or specified to a particular pain type, such as NPSI and BPI. Also, the direct association between pain and quality of life in these individuals show that pain could be an effective factor in reducing the quality of life.

The Cronbach's alpha coefficient and the inter-item correlation of the SF-MPQ indicated that this tool generally examines the concept of pain in patients with PD, which is in line with the results of previous studies in other populations [7, 8, 10–14]. The nearly acceptable Cronbach's alpha for SF-MPQ-2 subscales represents the internal integration between the domains of the scale. Further, the results of this study showed high test-retest reliability for the total score of the SF-MPQ-2 in people with PD, which is similar to previous studies of this questionnaire in other disease entities [16, 17, 20, 21, 37]. Moreover, the SEM score indicates that this questionnaire is accurate enough to measure pain.

The majority of our study population was in H&Y stages of 1 and 2 which means low severity of PD; therefore, we might have fewer patients with severe pain, and this could be considered as a limitation of the study. The authors of this study recommend addressing this issue to extrapolate the results to future studies. Moreover, longitudinal studies are required to assess the sensitivity of the SF-MPQ-2 to measure the change in pain severity over time in patients with PD.

## 7. Conclusion

The results of this study suggest that the Short-Form McGill Pain Questionnaire-2 has acceptable reliability and validity for assessment of pain in Parkinson's disease population. The results of this study also showed that the application of this scale into the clinics and decision making in treatment needs further research which could be noted in future studies.

## Figures and Tables

**Table 1:** Demographic and clinical characteristics of individual with Parkinson disease (PD) (*n* = 428).

264 (62%)	Male	Sex (*n*, %)
164 (38%)	Female	

60.91 (11.44)	Age, years, mean (SD)	

5.39 (5.50)	Time since PD diagnosis, years, mean (SD)	

201 (46.96%)	Stage 1 (n,%)	Hoehn and Yahr PD stage
149 (34.81%)	Stage 2 (n,%)	
36 (8.50%)	Stage 3 (n,%)	
42 (9.81%)	Stage 4 (n,%)	

**Table 2:** Factor analysis for SF-MPQ-2 in people with idiopathic Parkinson's disease (*n* = 428).

Items	Factor 1	Factor 2	Factor 3	Factor 4
Throbbing pain	0.78	0.22	0.05	0.28
Cramping pain	0.81	0.14	0.20	0.02
Gnawing pain	0.71	0.31	0.34	0.16
Aching pain	0.54	0.19	0.35	0.15
Heavy pain	0.68	0.21	0.06	0.33
Tender	0.72	0.21	0.19	0.39
Shooting pain	0.17	0.78	0.21	0.09
Stabbing pain	0.32	0.72	0.17	0.08
Sharp pain	0.44	0.69	0.06	0.12
Splitting pain	0.12	0.79	0.09	0.39
Electric-shock pain	0.23	0.38	0.52	0.31
Piercing	0.56	0.22	0.65	0.18
Cold-freezing pain	0.02	0.49	0.51	0.18
Hot-burning pain	0.40	0.38	0.46	0.15
Pain caused by light touch	0.24	0.11	0.59	0.28
Itching	0.11	0.25	0.68	0.17
Tingling or ‘pins and needles'	0.50	0.08	0.53	0.12
Numbness	0.33	0.06	0.74	0.07
Tiring-exhausting	0.14	0.45	0.14	0.53
Fearful	0.28	0.08	0.38	0.74
Sickening	0.28	0.25	0.15	0.79
Punishing-cruel	0.30	0.47	0.24	0.53

**Table 3:** Correlation between four scales and SF-MPQ-2 in people with idiopathic Parkinson's disease (*n* = 428).

SF-MPQ-2	DN4	KPPS	BPI		VAS-pain	NPSI	PDQ-8
			Interference	Severity			
Continuous	0.45	0.65	0.70	0.72	0.64	0.77	0.29
Intermittent	0.36	0.44	0.44	0.48	0.39	0.53	0.20
Neuropathic	0.56	0.61	0.66	0.65	0.57	0.85	0.29
Affective	0.31	0.43	0.55	0.44	0.40	0.55	0.28
Total score	0.55	0.70	0.74	0.74	0.68	0.85	0.32

DN4: Douleur neuropathic 4. KPPS: King's Parkinson's disease pain scale. BPI: Brief pain inventory. VAS-pain: Visual analog scale-pain. SF-MPQ-2: Short-form McGill pain Questionnaire-2. NPSI: Neuropathic pain symptom inventory. PDQ-8: Parkinson's disease Questionnaire-8. ^*∗∗*^All tests were statistically significant *p* ≤ 0.001.

**Table 4:** Interitem Correlation for SF-MPQ-2 in people with idiopathic Parkinson's disease (*n* = 428).

Items	1	2	3	4	5	6	7	8	9	10	11	12	13	14	15	16	17	18	19	20	21	22
1. Throbbing pain	1																					
2. Shooting pain	0.66	1																				
3. Stabbing pain	0.61	0.61	1																			
4. Sharp pain	0.53	0.48	0.54	1																		
5. Cramping pain	0.25	0.38	0.30	0.24	1																	
6. Gnawing pain	0.48	0.40	0.50	0.48	0.22	1																
7. Hot-burning pain	0.48	0.48	0.57	0.47	0.39	0.39	1															
8. Aching pain	0.44	0.50	0.48	0.46	0.38	0.41	0.45	1														
9. Heavy pain	0.27	0.38	0.37	0.30	0.50	0.41	0.42	0.44	1													
10. Tender	0.37	0.44	0.42	0.60	0.30	0.67	0.52	0.41	0.41	1												
11. Splitting pain	0.40	0.30	0.37	0.60	0.29	0.49	0.45	0.30	0.23	0.67	1											
12. Tiring-exhausting	0.49	0.62	0.46	0.35	0.43	0.35	0.42	0.42	0.50	0.38	0.24	1										
13. Sickening	0.50	0.34	0.40	0.36	0.23	0.71	0.31	0.33	0.33	0.50	0.49	0.43	1									
14. Fearful	0.57	0.40	0.40	0.63	0.27	0.51	0.50	0.28	0.25	0.69	0.74	0.39	0.58	1								
15. Punishing-cruel	0.32	0.39	0.44	0.44	0.54	0.29	0.42	0.40	0.27	0.44	0.35	0.50	0.21	0.39	1							
16. Electric-shock pain	0.52	0.44	0.55	0.43	0.37	0.48	0.47	0.57	0.46	0.50	0.37	0.37	0.48	0.44	0.39	1						
17. Cold-freezing pain	0.24	0.21	0.22	0.37	0.43	0.27	0.37	0.26	0.33	0.38	0.48	0.30	0.31	0.47	0.33	0.32	1					
18. Piercing	0.50	0.53	0.68	0.64	0.33	0.33	0.61	0.47	0.30	0.58	0.48	0.45	0.27	0.57	0.62	0.49	0.38	1				
19. Pain caused by light touch	0.52	0.53	0.51	0.44	0.27	0.41	0.31	0.46	0.31	0.33	0.45	0.36	0.40	0.46	0.30	0.49	0.30	0.45	1			
20. Itching	0.63	0.55	0.44	0.34	0.33	0.32	0.47	0.40	0.28	0.31	0.26	0.51	0.37	0.42	0.28	0.53	0.37	0.48	0.47	1		
21. Tingling or ‘pins and needles'	0.52	0.51	0.43	0.40	0.46	0.35	0.51	0.45	0.40	0.30	0.27	0.42	0.31	0.33	0.35	0.46	0.34	0.39	0.39	0.53	1	
22. Numbness	0.36	0.36	0.43	0.25	0.56	0.31	0.47	0.35	0.50	0.24	0.22	0.41	0.30	0.26	0.43	0.52	0.34	0.43	0.32	0.37	0.63	1

## Data Availability

The data used to support the findings of this study are included in the article.
